# Training an Embedded Workforce to Realize Health System Impacts and the Promise of Learning Health Systems: Comment on "Early Career Outcomes of Embedded Research Fellows: An Analysis of the Health System Impact Fellowship Program"

**DOI:** 10.34172/ijhpm.8667

**Published:** 2024-09-16

**Authors:** Elizabeth M. Yano

**Affiliations:** ^1^VA HSR Center for the Study of Healthcare Innovation, Implementation & Policy (CSHIIP), VA Greater Los Angeles Healthcare System, Los Angeles, CA, USA.; ^2^Division of General Internal Medicine and Health Services Research, Department of Medicine, UCLA Geffen School of Medicine, Los Angeles, CA, USA.; ^3^Department of Health Policy & Management, UCLA Fielding School of Public Health, Los Angeles, CA, USA.

**Keywords:** Learning Health Systems, Embedded Research, Implementation Science, Training Programs, Research Impacts

## Abstract

Learning health systems (LHSs) are designed to systematically integrate external evidence of effective practices with internal data and experience to put knowledge into practice as a part of a culture of continuous learning and improvement. Researchers embedded in health systems are an essential component of LHSs, with defined competencies. However, many of these competencies are not generated by traditional graduate/post-graduate training programs; evaluation of new LHS training programs has been limited. This commentary reviews and extends results of an evaluation of early career outcomes of fellows in one such program designed to generate impact-oriented career pathways embedded in healthcare systems. Discussion considers the need for increasingly rigorous evaluation methods to ensure production of high-quality professionals ready for system engagement, the importance of training and preparing other LHS stakeholders as effective partners and evidence users, and the promise and challenges in advancing the science and practice of embedded research in LHSs.

 The return on investment of health research has long been challenged by the “voltage drop” in outcomes from clinical research focused on demonstrating efficacy to that actually realized in actual healthcare settings and experienced by their patients.^1^ This “research to real world” knowledge gap is at the heart of efforts to lift up and further build health systems research and implementation science capacity and ensure their relevance and impacts through partnerships with healthcare delivery systems.^2^ The promise of learning health systems (LHSs), where such multilevel and engaged partnerships are organized and supported, is in their ability to use research and evaluation evidence linked to operational priorities to transform care and outcomes.^3^

 While LHS competencies have been defined, multifaceted programs described, and career trajectories postulated,^4,5^ little is known about effective strategies for training this next generation of applied and embedded scholars. Kasaai et al, recently reported on the outcomes of just such a program, focused on early career trajectories of postdoctoral research fellows after participation in a Health System Impact Fellowship.^6^ The Fellowship was designed to confer LHS competencies prioritized by potential employers (eg, leadership, change management, negotiation) through relevant didactics, experiential training embedded in a health system (organized around a project of high priority to the organization), and co-mentorship from a university-based academic and health system leader, in the context of a 2-year cohort-based approach to training.^6^ Overall, they found that all graduated fellows were employed and in diverse sectors (eg, academic, public, healthcare delivery, and private sectors), chiefly in alignment with their career aspirations at program onset. Self-reported career preparedness and readiness were high.

 Evaluations of efforts to build embedded research competencies in service of achieving LHS outcomes are critical at the same time they raise important questions (Figure 1). In Kasaai et al, full employment post-fellowship was laudable, as was trainee multisectoral distribution, and yet the majority ended up in academia. The absence of prior employment patterns and comparison groups makes it difficult to know if their results are attributable to an existing time trend (no program effect), a positive programmatic outcome (as suggested), or worse, a program failure, if the goal was to primarily embed graduates in delivery systems. Consistency between initial career aspirations and employment outcomes is similarly challenging to interpret. While only about half of fellows who aspired to become an embedded researcher did so, the program’s focus on multidisciplinary collaboration, health system embedding, and networking may have yielded opportunities not previously considered. Academic mentors may also have lacked embedded research experience themselves, so fellows may have modeled themselves toward academia, whereas being health system leaders (who served as co-mentors) was neither a career aspiration nor expected program outcome. Information on curricular details (eg, appropriate theories, systems science, implementation science, policy evaluation) and engagement content, quality, or time spent by different mentor types was not available, though these graduates likely blazed new trails across sectors, armed with skills and experience not common among their predecessors. To the authors’ credit, they acknowledged many of their evaluation limitations, including the lack of comparison group, small sample size, reliance on online data sources, and limited time since graduation (2-4 years). Training impacts need to be assessed for longer term career outcomes (eg, retention, promotion, career trajectories), traditional indicators of productivity (eg, grants funded, papers published), and very importantly, on the health systems that hire them (eg, operations projects approved, levels of multilevel engagement achieved, impacts on care delivery, patient outcomes, cost savings). The authors also noted a significant loss to follow-up for post-program surveys, which could have contributed to bias in measures of readiness, preparedness, and satisfaction with supervisors’ support of their career prospects. Less attention was paid to the need for more validated measures, lack of a mixed methods approach, and a more rigorous post-program survey that might have illuminated what aspects of the program fellows thought were most effective. Information about the nature of the projects that fellows designed and conducted in their respective health systems would also be useful in gauging the quality and caliber of their embedded work, in addition to the multilevel engagement and partnerships they garnered and the outcomes they achieved during their relatively brief tenures. Evaluation of target health system contexts is also key, including the level of LHS implementation, availability of organizational supports to conduct embedded research, and the cultural milieu that either helps or hinders the kinds of learning loops and multilevel stakeholder engagement that differentiates these kinds of models.^7^ Evaluation of their competencies in practice from their new employers would also be an important contribution, as would assessments of their training needs over the long term, while also tracking their mentorship of the next generation of embedded researchers.^5^ The challenge is how to obtain adequate funding to pursue rigorous evaluations that address these and other methodological considerations on the path to improved outcomes of embedded research and furthering the business case for adoption and effective integration of embedded researchers.^8^

**Figure 1:**
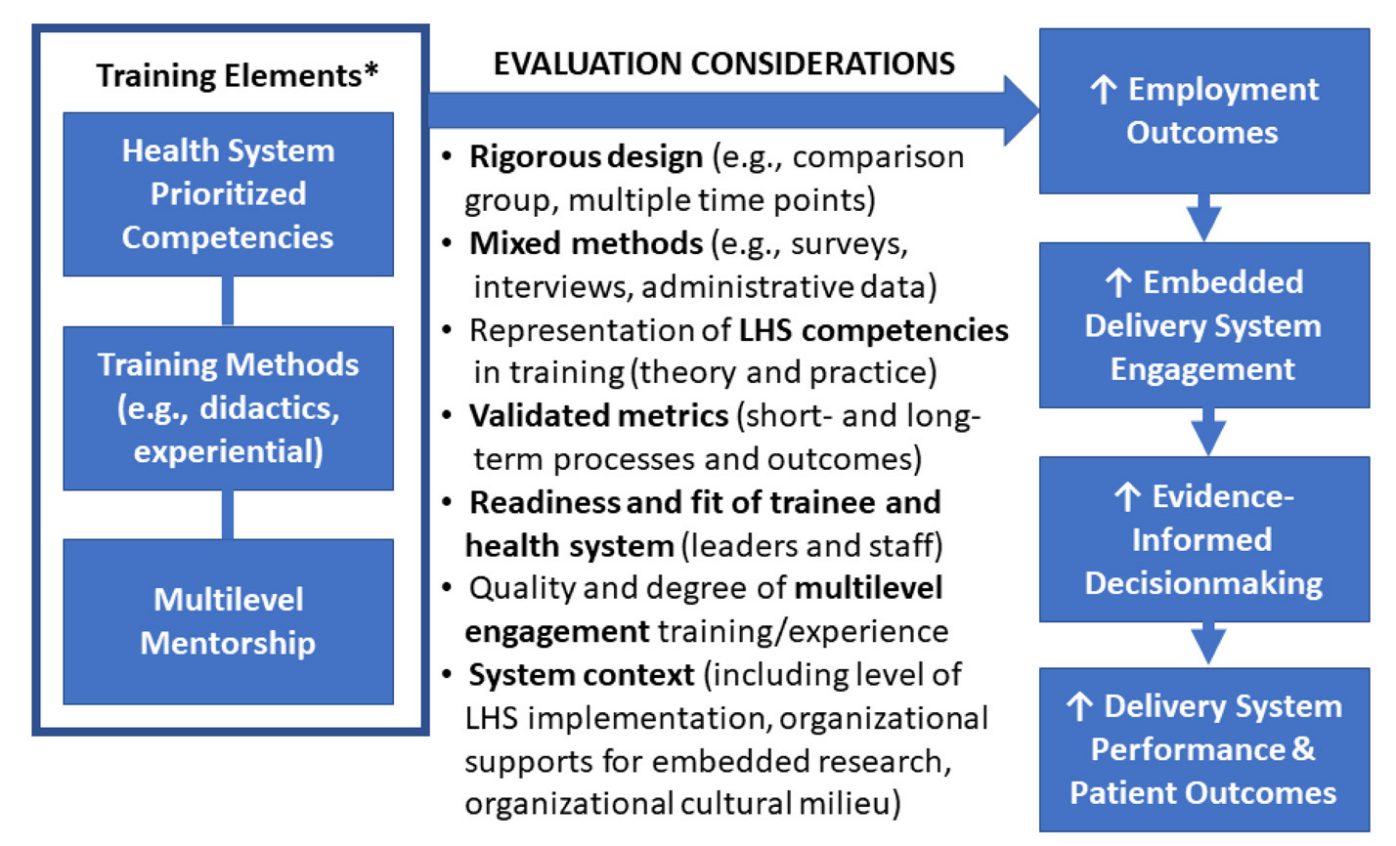
Evaluating Embedded Research Competencies and Outcomes in a Learning Health System Context. * Adapted from Kasaai et al^6^ (eg, employer-prioritized broadened to health system, two-level mentorship—academic and senior health system leader—broadened to all levels). Abbreviation: LHS, Learning health system.

 Advancing LHS functions and tenets is obviously not just about building embedded research capacity and will require going far beyond training researchers to work effectively in health systems, especially given how variably health systems have actually adopted essential LHS elements.^9^ Instead, the path to LHS adoption and maturity requires reciprocally priming the health system by building and supporting the necessary infrastructure, processes, and training (eg, numeracy) to be an effective “host” if not “receptor” for embedded researchers (ie, organizational symbiosis, co-production, synergies), ideally where the whole is greater than the sum of its parts.^10^ Health system leaders and managers need training on how to pose questions to their own data systems with and through embedded staff, how to appraise evidence worth implementing (ie, when is there enough evidence that warrants changing existing practice to new routines), how to balance evidence and improvement value in relation to the costs of change (and potential costs of inertia), and how to optimally integrate embedded researchers in health system workflows and operations. The extent to which employers and staff are themselves ready for LHS implementation and engagement with embedded researchers is not known, though experience suggests substantial work is needed on all levels of health system organizations.

 While, in some respects, the business case for training and employing embedded researchers in a LHS has never been clearer, initiatives that clearly and consistently communicate their value to stakeholders within and outside the health sector are needed.^8^ Some of the push-pull to adequately adopt LHS functions in health system organizations may be a result of historical failures of in-house continuous quality improvement initiatives to achieve hoped-for improvements in care.^11^ In contrast, *evidence-based* quality improvement, where researchers partner with health system leaders is an effective embedded research strategy, which may confer greater impacts if not a competitive advantage in enhancing LHS functions (eg, self-efficacy implementing new care models, change-readiness, team-based communication, team function), while also reducing burnout.^12,13^ Better training programs for all LHS stakeholders are essential, but at the core of some of these challenges is the persistent lack of a shared language, common frameworks, aligned incentives, and meaningful multilevel and interdisciplinary engagement anchored in diverse contexts of health systems and the patients they serve.^2,7^

 Ultimately, the science of embedded research needs to more strongly meld with the pragmatics and evidence needs of health system practice and policy in an LHS context. Nowhere is that more apparent than in starting with the nature of the roles between health system decision-makers and researchers. Historically, research has been almost exclusively in the purview of university-based academic faculty who may study a system and whose external vantage point has been lauded as central to objective scientific inquiry as independent agents of “truth.” However, this model of external evidence generation, while broadly useful and important in its own right, typically treats decision-makers as passive recipients of evidence rather than partners in agenda-setting let alone real-world application of evidence-based improvements, thus limiting the potential for LHS development let alone self-reliance. In contrast, the US Department of Veterans Affairs (VA) has focused on an embedded research-operations partnership model, with an intramural research program aligned with system priorities and Veteran needs.VA health systems researchers, many of whom also have academic affiliations, are oriented to the importance of operations leader engagement before, during, and after each project. Funding may be predicated on system partners demonstrating commitment to implementing research results, while Veteran engagement is also a scored element.Many national VA program offices overseeing practice and policy in specific clinical and policy areas (eg, primary care, women’s health, health equity) also directly fund evaluation, frequently relying on the embedded research workforce given their training, experience, system knowledge (eg, many also deliver VA clinical care), and attention to contexts of the system, its providers and staff, and patients, moving discovery to system-wide change.^14^ In yet another model, the World Health Organization (WHO) has invested in an initiative that actually requires decision-makers to be the research leaders, given their system-level authority over practice and policy; early evidence suggests promising outcomes across diverse international exemplars, though such models may require an uncommon degree of research acumen among system leaders.^7^ Both partnership models, as well as many others underway, require recognition and optimization of the roles of researchers as sources of independent and objective evidence, including when that evidence highlights problems. Lessons across these and other diverse LHS experiments suggest the vital importance of recognizing the value of (1) embedded researchers trained and supported to be responsive to system and patient needs, (2) their routine engagement of and collaboration with multilevel decision-makers, as well as frontline providers and staff, in all aspects of embedded research, (3) their explicit attention to community and system contexts in the design, conduct, and use of research and data, with evidence and communication/coordination tailored to local needs, and (4) an embrace of cyclical knowledge generation and use of rigorous, practice-based evidence in deploying innovations and implementing evidence-based practices.^7,14^

 In reality, it is very difficult to achieve the tenets of an LHS without embedded researchers, and their value proposition spans broad LHS principles^3^ (eg, science, informatics, incentives, culture) (Figure 2). For *science*, they serve as enablers of high-quality evidence generation, rigorous evaluation, and implementation of evidence-based practice, while also serving as internal experts and trainers of other LHS stakeholders. For *informatics*, their training may help ensure health systems rely on valid data and continuously improve encounter data, while adding other data (eg, provider/staff, organizational, area data) that refine the questions decision-makers may pose and researchers may help answer. Others may be able to advance data tools and electronic health record based improvements at the point of care or for practice-wide improvement, while their role in improved statistical modeling and machine learning will enhance health systems’ ability to explore the value of artificial intelligence in improving care quality and efficiency. For *incentives*, some may be anchored in traditional markers toward academic promotion (eg, funding, peer-reviewed papers) but others likely reflect the culture of embedded researchers to have their research make a difference on system performance. And, for *culture*, embedded researchers often become central to an array of improvement initiatives, supporting rapid cycle improvement, adapting evidence to local contexts, and enhancing collaboration in ways that empower teams and lowers burnout.^13^ When researchers are integral to the delivery system, enhancing productive interactions with leaders and frontline providers and staff alike, within and across care delivery settings, they begin to embody the iterative clinical and research lifecycle that is central to LHS functions.

**Figure 2:**
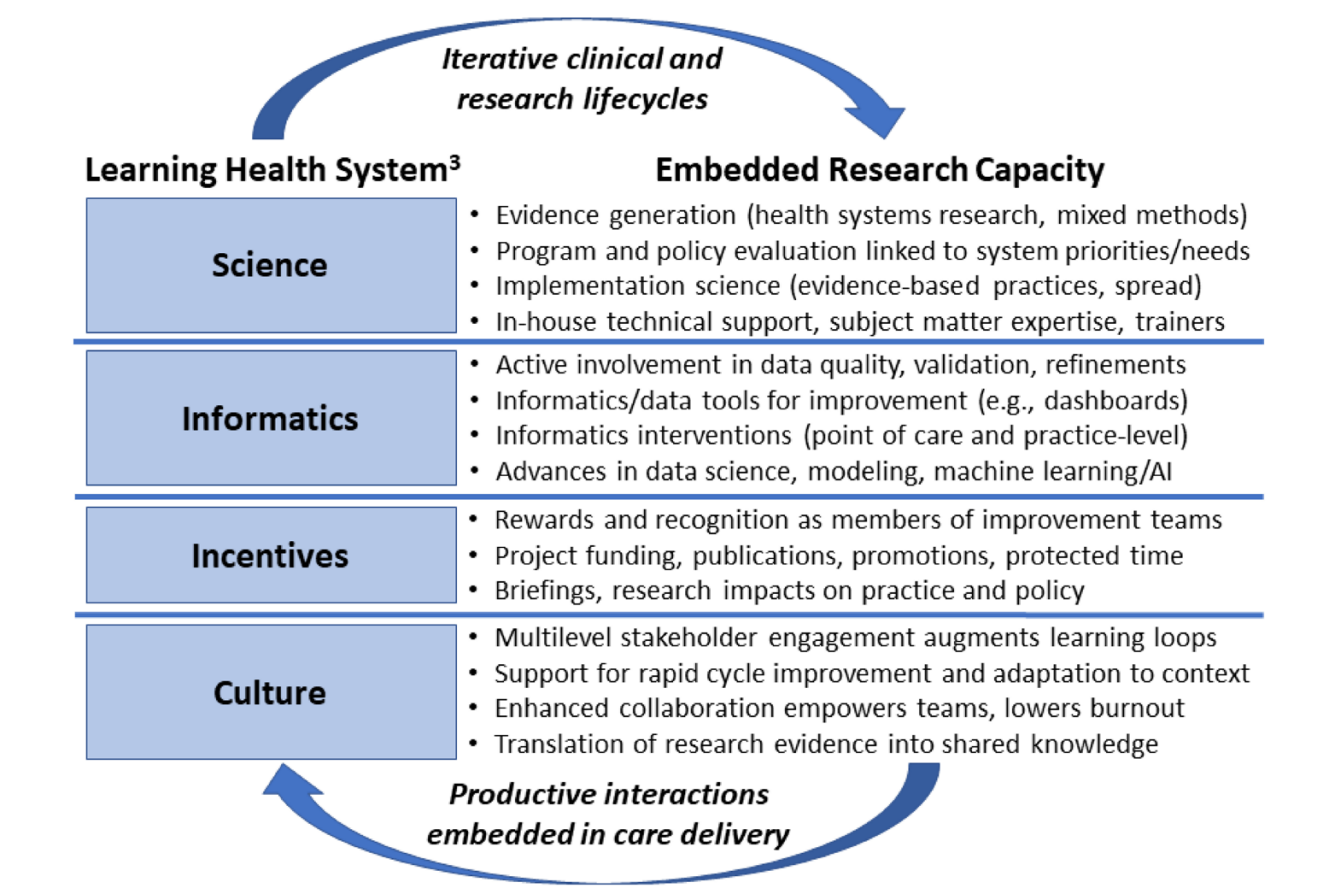
Embedded Research Capacity in Learning Health Systems. Abbreviation: AI, artificial intelligence.

 While the relevant literature in this area has grown at a fast pace, increasingly drawing from diverse disciplines and fields of inquiry (eg, community engaged research, participatory action research, learning theories, systems science, informatics/data science, engagement science, implementation science, policy evaluation),^4,7,8^ rigorous evaluation of novel training approaches that take these lessons and needs into account and explore different models of embedded research in support of advancing LHSs remain critical to advancing the field.^15^

## Ethical issues

 Not applicable.

## Conflicts of interest

 Author declares that she has no conflicts of interest.

## Disclaimer

 The views expressed in this article are those of the author and do not necessarily reflect the position or policy of the US Department of Veterans Affairs or the US Government.
